# Familial congenital cyanosis caused by Hb-M_Yantai_(α-76 GAC → TAC, Asp → Tyr)

**DOI:** 10.1590/S1415-47572010005000058

**Published:** 2010-09-01

**Authors:** Yanbo Sun, Pingyu Wang, Youjie Li, Fei Jiao, Zunling Li, Ying Ma, Wei Li, Shuyang Xie

**Affiliations:** 1 , The 107th Hospital of PLA and The Affiliated Hospital to Bin Zhou Medical UniversityP.R. China; 2 Department of Biochemistry and Molecular Biology, Bin Zhou Medical UniversityP.R. China; 3 , Wenzhou Medical CollegeP.R. China

**Keywords:** congenital cyanosis, hemoglobin, methemoglobinemia, a-globin gene, China

## Abstract

Methemoglobin (Hb-M) is a rare hemoglobinopathy in China. We hereby report on a family living in Yantai, East China, with congenital cyanosis due to Hb-M mutation. The proband, a 65-year-old female, presented 63% oxygen saturation. Both Hb-M concentration and arterial oxygen saturation remained unchanged, even following intravenous treatment with methylene blue. There was also no change in blood-color (chocolate-brown) after adding 0.1% KCN. A fast-moving band (Hb-X) in hemolysates was found by cellulose acetate electrophoresis, the Hb-X/Hb-A ratio exceeding 10%. GT transition at 131nt of exon 2, although present in one of the α_2_ -globin alleles, was not found in α_1_ -globin alleles as a whole. This mutation leads to the aspartic acid to tyrosine substitution (Asp76Tyr). In this family, the novel mutation in the α_2_ -globin gene resulted in a rare form of congenital cyanosis due to Hb-M. This hemoglobin was named Hb-M _Yantai_ .

Congenital cyanosis can be caused by hereditary methemoglobinemia due to either NADH-methemoglobin reductase (NADH-MR) deficiency (Percy and Aslan, 2008; Percy and Lappin, 2008), or the presence of an abnormal hemoglobin (Hb-M) (Da-Silva *et al.*, 2003; Kedar *et al.*, 2005). Mutations in either α- or β-globin genes have been described as the cause of Hb-M disorders (Burkert *et al.*, 1976; Orisaka *et al.* 1995, Ameri *et al.*, 1999; Kedar *et al.*, 2005).

In this report, we describe a novel mutation in the α_2_ globin gene, which caused non-debilitating congenital methemoglobinemia in a family, with cyanosis as the only obvious manifestation.

The family came from Yantai, Shan-Dong Province, China. Informed consent, blood samples and clinical evaluation were obtained from all participating family members, under protocols approved by the Institutional Review Board of the 107th Hospital of PLA and the hospital affiliated to Bin Zhou Medical University.

Red blood cell (RBC) count, Hb, mean corpuscular volume (MCV), reticulocyte counts and mean corpuscular Hb (MCH) were determined using a Hematology Analyzer (KX-21, Sysmex, Japan). An oximeter was employed for detecting methemoglobinemia. Hb-M, expressed as a percentage of Hb, was estimated using a modified Evelyn and Malloy method (Davidson and Henry, 1969). In brief, hemolysates were treated with potassium ferricyanide [K_3_Fe(CN)_6_] and absorbance measured from 400 to 700 nm. Hb-M reductase catalyzed the NADH-linked reduction of several substrates, including ferricyanide. The activity of cytochrome b5 reductase was measured spectrophotometrically by monitoring NADH oxidation (via ferricyanide reduction) at 340 nm (Haymond *et al.*, 2005). Hemoglobin electrophoresis was performed on cellulose acetate (Elderdery *et al.*, 2008), and the ratio of a fast-moving band (Hb-X) relative to Hb-A was defined using AlphaEaseFC software (Alpha Innotech, USA).

DNA, extracted from blood cells by standard phenol/chloroform extraction methods, was used as a template for PCR amplification to detect possible mutations. The β-globin gene was amplified by primers β_1_ and β_2_, with a program consisting of 28 cycles of denaturation at 94 °C for 45 s, annealing at 58 °C for 45 s and elongation at 72 °C for 120 s, in an Eppendorf cycler. The α-globin gene was amplified by primers α_forward_ and α_reverse_, which amplify both α_1_-globin and α_2_-globin. The PCR amplification consisted of 30 cycles of denaturation at 94 °C for 60 s, annealing at 56 °C for 60 s, and elongation at 72 °C for 90 s. The PCR products were then cloned into a T vector (Promega) to construct T-α. The sequences of inserted DNA from 9-10 clones of each amplified DNA sequence were determined by using an automatic DNA sequencer (Biosune, Shanghai, China). Specific primers were designed for amplifying α_1_- and α_2_-globin genes, whether carrying or not the detected mutation. The PCR amplification consisted of 28 cycles of denaturation at 94 °C for 30 s, annealing at 55 °C for 45 s, and elongation at 72 °C for 30 s. The primers are shown in Table 1.

The proband was a 65-year-old female (Figure 1A, IV7) seeking medical treatment for progressive fatigue and a two days long headache. She presented marked cyanosis, and so was intravenously treated with methylene blue at a dose of 1.5 mg/kg body weight. However, the cyanosis, Hb-M concentration and arterial oxygen saturation remained unchanged. The patient was moderately obese. Blood pressure was 135/85 mmHg, pulse 82 beats/min, respiratory rate 17 breaths/min and temperature 37.6 °C. Lymphadenopathy and hepatosplenomegaly were nonexistent. The patient's medical history revealed no evidence of cardiopathy or exposure to drugs or chemicals. Her son and two daughters (Figure 1A, V5-7) were all in good health. Family history showed no evidence of anemia. She was initially diagnosed with influenza. Although one week later there was an improvement, cyanosis persisted. As shown by gastroendoscopy, her lips, hands and stomach were cyanotic (Figure 1B), thereby indicating influenza not to be the cause. Although otherwise healthy, 14 members of her family presented this ailment (Figure 1A).

The results of hematological analysis of the proband and family members, as well as health controls, can be seen in Table 2. Oxygen saturation of the proband, IV10, V10 and V12 was much lower than that of the other three healthy individuals (95%-100%). Blood-color change after exposing 0.2 mL of blood to 1-3 drops of 1% KCN was also assessed, thereby revealing no change in any patient.

**Figure 1:**
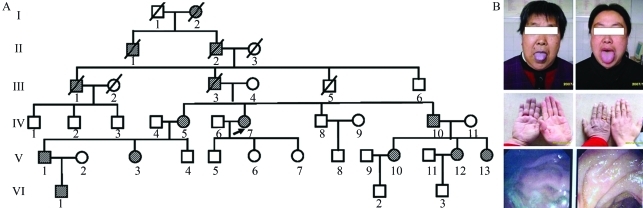
(A) Family pedigree; grey-striped symbols are family members that present HB-M disease with cyanosis. (B) Proband with cyanosis (left) and one of her noncyanotic daughters (V6; right): lips, hands and stomach visualized by gastroendoscopy.

Approximately 5 g/dL of reduced Hb is required to produce cyanosis in disorders involving deoxygenated Hb. However, 1.5 g/dL of Hb appears to be required in disorders involving nonfunctional hemoglobin (Griffey *et al.*, 2000). Surprisingly, in this study Hb concentration was not markedly less. However, cellulose acetate electrophoresis revealed a fast-moving band (Hb-X or Hb-M) ahead of the Hb-A band in the hemolysates of patients IV7, IV10, V10 and V12 (Figure 2A). The Hb-X/Hb-A ratios (Figure 2A) for these patients were 11.1 ± 0.8%, 13.0 ± 0.4%, 13.0 ± 1.2% and 15.0 ± 0.8%, respectively, much higher than those of the healthy control, as well as those of V5, V6 and V7 (< 1%). Our results showed that an abnormal Hb-X/Hb-M ratio was about 13.0% (about 1.5 g/dL) in such patients, thereby possibly giving rise to the clinical features of cyanosis.

**Figure 2:**
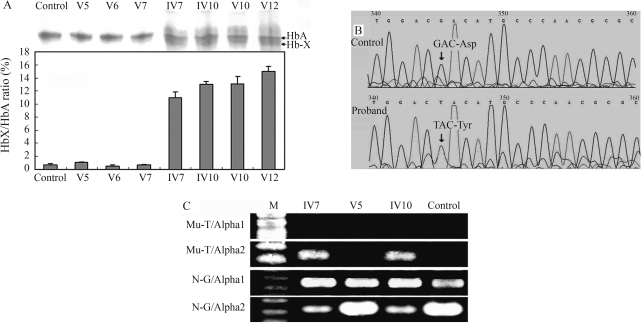
Hb and mutation analyses. (A) A fast-moving band, Hb-X, detected in cyanotic individuals after cellulose acetate electrophoresis; Hb-X/Hb-A ratios, as determined using AlphaEaseFC software. (B) Chromatograms of the sense-strand sequence from the patients showing the GT mutation. (C) Location of the GT mutation in _2_ globin genes by PCR (see Table 2 for primers). M: 100 bp DNA marker.

The detection of Hb-X prompted us to search for mutations in α- and β-globin genes. None were found in the latter (data not shown). The GT substitution was found at 131 nt of exon 2 in one of the α_2_-globin alleles in the proband and in her cyanotic relatives IV7, IV10, V10 andV12 (Figure 2B and C), but not at 131 nt of exon 2 in any α_1_-globin allele. This mutation leads to amino acid substitution (Asp76Tyr). Aspartic acid is an acidic hydrophilic amino acid, whereas tyrosine is a non polar hydrophobic amino acid. The altered physicochemical properties may cause structural change in the α_2_-globin chain.

The presence of cyanosis in this six-generation pedigree demonstrates dominant trait inheritance. Our results pointed to a GT substitution in α_2_-globin genes in those members of the family with cyanosis, although not occurring in noncyanotic relatives, as being the causative trait. We designated the detected Hb-M variant as Hb-M_Yantai_.

## Figures and Tables

**Table 1:** Oligonucleotide primers

Primer	Sequence	Amplified segments
β_1_	5'-AGGGCAGAGCCATCTATT-3'	β-globin gene (1774 bp)
β_2_	5'-CAGCCTCACCTTCTTTCA-3	
α_forward_	5'-CGGCACTCTTCTGGTCCC-3'	α_1_-globin and α_2_-globin r (691 bp)
α_reverse_	5'-CTTGTCCAGGGAGGCGTG-3'	
Mu-T	5'-GTGGCGCACGTGGACT-3'	Mutated α_1_-globin gene (235 bp)
α_1_-only	5'-GCAGAGAAGAGGGTCAGTGG-3'	
Mu-T	5'-GTGGCGCACGTGGACT-3'	Mutated α_2_-globin gene (228 bp)
α_2_-only	5'-GCAGAGAAGAGGGTCAGTGC-3'	
N-G	5'-GTGGCGCACGTGGACG-3'	Normal α_1_-globin gene (235 bp)
α_1_-only	5'-GCAGAGAAGAGGGTCAGTGG-3'	
N-G	5'-GTGGCGCACGTGGACG-3'	Normal α_2_-globin gene (228 bp)
α_2_-only	5'-GCAGAGAAGAGGGTCAGTGC-3'	

**Table 2:** Hematological and enzyme analysis.

Parameter	Healthy control	Family Members*						
		V5	V6	V7	IV7**	IV10**	V10**	V12**
Hb (g/dL)	11.6-17.9	15.1	14.4	14.3	14.4	14.9	14.2	14.0
HCT (%)	37-52	46.4	43.8	43.2	45.6	46.2	45.5	42.1
RBC (x 10^6^/μ L)	3.5-5.7	4.73	4.82	4.47	4.72	4.94	4.32	4.85
WBC (x 10^3^/μ L)	4-10	7.5	5.52	4.57	6.27	6.23	5.41	6.02
MCV (fl)	80-98	98.1	90.9	96.6	96.6	93.5	94.2	95.4
MCH (pg)	26-35	31.9	29.9	32.0	30.5	30.2	29.4	31.1
MCHC (g/dL)	320-360	325	329	331	316	323	320	331
Reticulocytes (%)	0.5-1.5	1.1	0.6	1.1	1.2	1.4	1.2	1.1
Oxygen saturation (%)	95-100	98	98	100	63%	56%	65%	61%
Hb-M (%)	0.5	0.4	0.2	0.6	1.0	0.7	0.9	0.8
Spectroscopic analysis absorption maxima (nm)	500/630	500/630	500/630	500/630	500/630	500/630	500/630	500/630
Cytochrome b5 reductase activity (normal 18.7 ± 3.5 U/g)	19.6	20.7	19.7	20.5	21.2	19.6	18.4	20.7

*Pedigree in Figure 1A; IV7, Proband ; **Cyanotic individuals.

## References

[Amerietal1999] Ameri A., Fairbanks V.F., Yanik G.A., Mahdi F., Thibodeau S.N., McCormick D.J., Boxer L.A., McDonagh K.T. (1999). Identification of the molecular genetic defect of patients with methemoglobin M-Kankakee, alpha87 (F8) His → Tyr: Evidence for an electrostatic model of alphaM hemoglobin assembly. Blood.

[Burkertetal1976] Burkert L.B., Sharma V.S., Pisciotta A.V., Ranney H.M., Bruckheimer S. (1976). Hemoglobin M equon beta 41 (C7) phenylalanine leads to tyrosine. Blood.

[DavidsonandHenry1969] Davidson I., Henry J.B. (1969). Clinical Diagnosis by Laboratory Methods.

[Da-Silvaetal2003] Da-Silva S.S., Sajan I.S., Underwood J.P. (2003). Congenital methemoglobinemia: A rare cause of cyanosis in the newborn - A case report. Pediatrics.

[Elderderyetal2008] Elderdery A.Y., Mohamed B.A., Karsani M.E., Ahmed M.H., Knight G., Cooper A.J. (2008). Hemoglobinopathies in the Sudan. Hemoglobin.

[Griffeyetal2000] Griffey R.T., Brown D.F., Nadel E.S. (2000). Cyanosis. J Emerg Med.

[Haymondetal2005] Haymond S., Cariappa R., Eby C.S., Scott M.G. (2005). Laboratory assessment of oxygenation in methemoglobinemia. Clin Chem.

[Kedaretal2005] Kedar P.S., Nadkarni A.H., Phanasgoankar S., Madkaikar M., Ghosh K., Gorakshakar A.C., Colah R.B., Mohanty D. (2005). Congenital methemoglobinemia caused by Hb-MRatnagiri (beta-63CAT → TAT, His → Tyr) in an Indian family. Am J Hematol.

[Orisakaetal1995] Orisaka M., Sasaki T., Kato J., Harano K., Harano T. (1995). Hb M-Iwate [alpha 87 (F8) His → Tyr]: Analysis of the genomic DNA and biosynthesis. Rinsho Byori.

[PercyandAslan2008] Percy M.J., Aslan D. (2008). NADH-cytochrome b5 reductase in a Turkish family with recessive congenital methaemoglobinaemia type I. J Clin Pathol.

[PercyandLappin2008] Percy M.J., Lappin T.R. (2008). Recessive congenital methaemoglobinaemia: Cytochrome b(5) reductase deficiency. Br J Haematol.

